# Getting ready to use control: Advances in the measurement of young children’s use of proactive control

**DOI:** 10.1371/journal.pone.0175072

**Published:** 2017-04-18

**Authors:** Sabine Doebel, Jane E. Barker, Nicolas Chevalier, Laura E. Michaelson, Anna V. Fisher, Yuko Munakata

**Affiliations:** 1 Department of Psychology and Neuroscience, University of Colorado—Boulder, Boulder, Colorado, United States of America; 2 School of Philosophy, Psychology, and Language Sciences, University of Edinburgh, Edinburgh, Scotland, United Kingdom; 3 Department of Psychology, Carnegie Mellon University, Pittsburgh, Pennsylvania, United States of America; Swinburne University of Technology, AUSTRALIA

## Abstract

A key developmental transition in executive function is in the temporal dynamics of its engagement: children shift from reactively calling to mind task-relevant information as needed, to being able to proactively maintain information across time in anticipation of upcoming demands. This transition is important for understanding individual differences and developmental changes in executive function; however, methods targeting its assessment are limited. We tested the possibility that Track-It, a paradigm developed to measure selective sustained attention, also indexes proactive control. In this task children must track a target shape as it moves unpredictably among moving distractors, and identify where it disappears, which may require proactively maintaining information about the target or goal. In two experiments (5–6 year-olds, Ns = 33, 64), children's performance on Track-It predicted proactive control across two established paradigms. These findings suggest Track-It measures proactive control in children. Theoretical possibilities regarding how proactive control and selective sustained attention may be related are also discussed.

## Introduction

Every day we exercise control. Whether refraining from eating a sugary treat while on a diet, staying alert during a long meeting, or switching between tasks to meet deadlines, we draw on control to achieve our goals. This skill, termed executive function (EF), improves dramatically in childhood [1–3] and predicts success in key domains of human functioning (e.g., academic, health, and wealth; [4, 5]). As such, there has been great interest in improving EF through training (see [1] for a review).

EF develops in multiple ways during childhood. With age, children become increasingly capable of inhibiting and shifting between competing responses [6,7], and manipulating information held in mind [8]. Children also use increasingly abstract representations to support EF [9, 10, 2], and EF skills may become increasingly differentiated in the course of development [11–14].

EF also varies in the temporal dynamics of how it is engaged [15, 16]. Between 5 and 6 years of age, children shift from engaging EF reactively, in the moment it is needed, to also engaging EF proactively, in anticipation of upcoming demands [17–21]. For example, a 4-year-old may interrupt an ongoing activity to put on a raincoat if she is getting wet, whereas a 6-year-old may proactively seek the raincoat to put on before heading outside.

**Fig 1:**
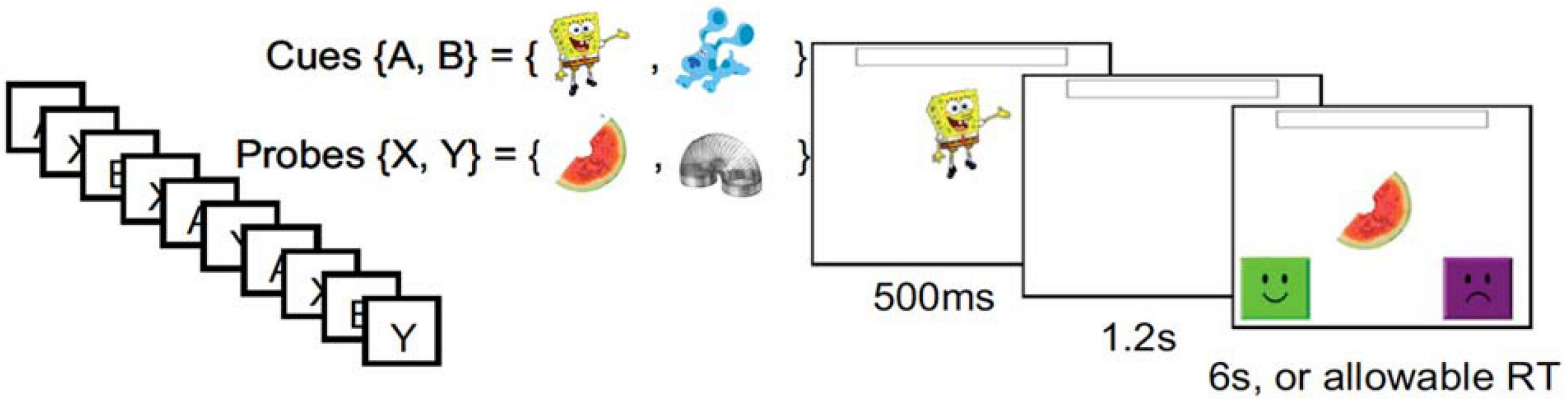
Child-adapted AX-CPT (reproduced from [18]). Children are presented with a cue (e.g., SpongeBob) followed by a probe (e.g., watermelon) and must provide the target (press happy face) or non-target response (press sad face), given the task rules. AX trials require the target response and occur 70% of the time, and other trials (AY, BX, BY) require the non-target response and each occur 10% of the time. Proactive control leads to longer RTs and more errors on AY relative to BX trials.

Evidence for this developmental transition in the proactive engagement of EF, termed ‘proactive control’, has been found using the AX Continuous Performance task (AX-CPT). This paradigm has been used extensively to measure reactive and proactive control in adults [22, 23, 16] and has been adapted for use with children [18, 21]. In this task, participants must provide a target response following the presentation of a specific probe (‘X’), but only if it is preceded by a specific cue (‘A’). On some trials the cue will be invalid (‘BX’ trials), or the probe will be invalid (‘AY’ trials), or both (‘BY’ trials). AX trials are frequent (typically 70% of all trials), creating a bias to select the target response when ‘A’ or ‘X’ appears (Fig 1). A proactive strategy is indicated by slowing and errors on AY relative to BX trials. Upon seeing the ‘A’ cue, proactive individuals should maintain it in mind and anticipate the ‘X’ probe and the need to provide a target response, which should in turn lead to slowing and errors on AY trials because this expectation is violated. A proactive strategy is also indicated by faster and more accurate responses on BX trials such that, upon seeing the ‘B’ cue, proactive individuals maintain it in mind and anticipate the need to provide the non-target response. (AX and BY trials could be aided by proactive or reactive control, or by simple prepotency in the case of AX trials, and so may be less informative.) Three-year-olds use a primarily reactive strategy, as evidenced by an absence of slowing on AY relative to BX trials and pupillometric indices indicating greatest effort during probe presentation [18]. Five- and 6-year-olds, on the other hand, show longer RTs on AY relative to BX trials, and greatest effort during the delay period between the cue and the probe, suggesting the use of proactive control [24, 21]. This difference between AY and BX trials increases with age, with 6-year-olds showing longer AY versus BX RTs than 5-year-olds, consistent with proactive control becoming more efficient during this age [21].

**Fig 2:**
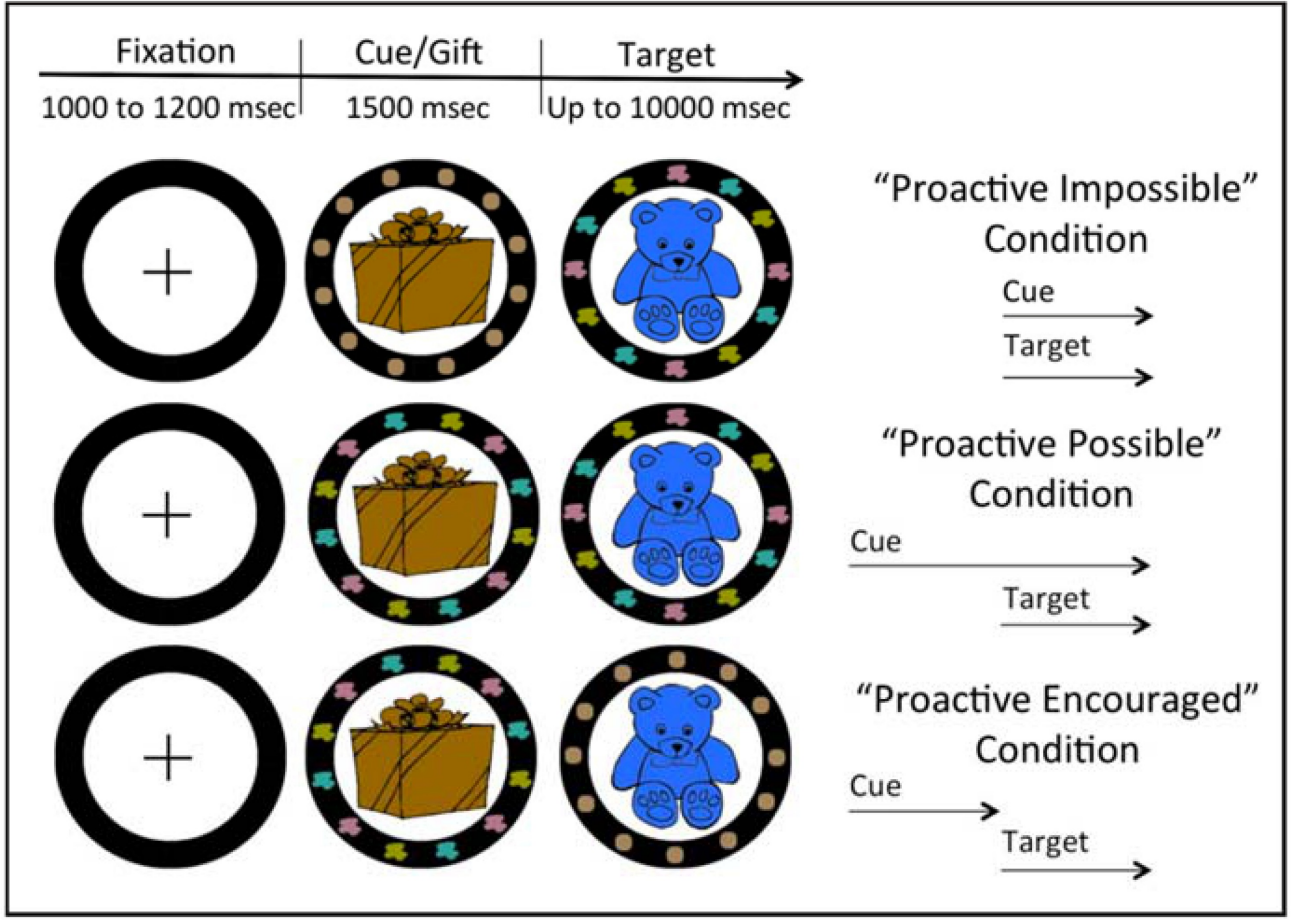
The cued task-switching paradigm (reproduced from [20]). Children switched between sorting the target (blue bear) by shape or by color. The patches of color within the black circle provided a cue that color is the relevant sorting dimension. In the Proactive-Impossible condition, the cue appeared at the same time as the target, making proactive preparation impossible (uninformative brown dots appeared in place of an informative cue, which were later replaced by the cue). In the Proactive-Possible condition, the task cue was presented prior to the target and remained visible when the target appeared, making proactive preparation possible but not critical. In the Proactive-Encouraged condition, the cue was presented prior to the target but disappeared before the target’s appearance, encouraging children to attend to and process the cue proactively.

Further evidence of this transition comes from a cued task-switching paradigm developed to assess whether children prepare in advance and the conditions that foster such preparation [20]. In this task, children are cued to sort bivalent stimuli (e.g., blue and red bears and cars) by shape or color, and, critically, the timing of the cue presentation is manipulated across three conditions (Fig 2). In the Proactive-Possible condition, the cue is presented prior to the presentation of the target stimulus, allowing children to prepare to sort by the indicated dimension before the target appears. In the Proactive-Impossible condition, the cue is presented at the same time as the target, such that children cannot prepare how to sort before the target appears. In the Proactive-Encouraged condition, the cue is presented in advance of the target but then disappears prior to the target’s appearance, making it more difficult to perform accurately without proactively maintaining the cue; this condition thus incentivizes children to adopt a more proactive strategy. Faster reaction times indicate that children engage proactive control. Ten-year-olds are faster in the Proactive-Possible and Proactive-Encouraged conditions compared to the Proactive-Impossible condition [20], suggesting that they prepare to sort by the indicated dimension whenever they can. In contrast, 5-year-olds are similarly slow in the Proactive-Impossible and Proactive-Possible conditions, suggesting that they tend to use a primarily reactive strategy of processing the cue once the target appears. However, they are significantly faster in the Proactive-Encouraged condition than in the other two conditions, suggesting that they can use proactive control when encouraged to do so [20].

Understanding this developmental transition in the temporal dynamics of control is critical to gaining insight into developmental mechanisms and individual differences in EF, and can inform interventions to improve EF; however, methodological limitations have constrained the progress of research on this topic. There are few established child measures of proactive control. Existing measures tend to be difficult to administer due to their length and complexity, limiting their use with young children or as part of larger task batteries. For example, AX-CPT includes numerous AX trials to establish a bias to select the target response upon seeing the ‘A’ cue, such that many trials are required to include sufficient numbers of the key trial types of interest (AY and BX). The cued task-switching paradigm requires numerous trials to assess reaction times reliably. In addition, both tasks have several rules, some complex, which children must master in a practice phase in order to advance to the test phase of the task. Apart from the need to develop measures that are more conducive for inclusion in assessment batteries, additional measures are also needed to address the task impurity problem: to target variance in task performance that is attributable to the construct of interest (e.g., proactive control) versus variance due to unrelated demands (e.g., perceptual processing and motoric responses). The task-impurity problem is particularly problematic for measuring executive functions and related constructs, which operate on other cognitive processes [25]. Developing additional measures that index proactive control but differ in their other demands can alleviate the impurity issue by allowing the formation of a composite measure or statistical extraction of a latent variable [25, 26].

**Fig 3:**
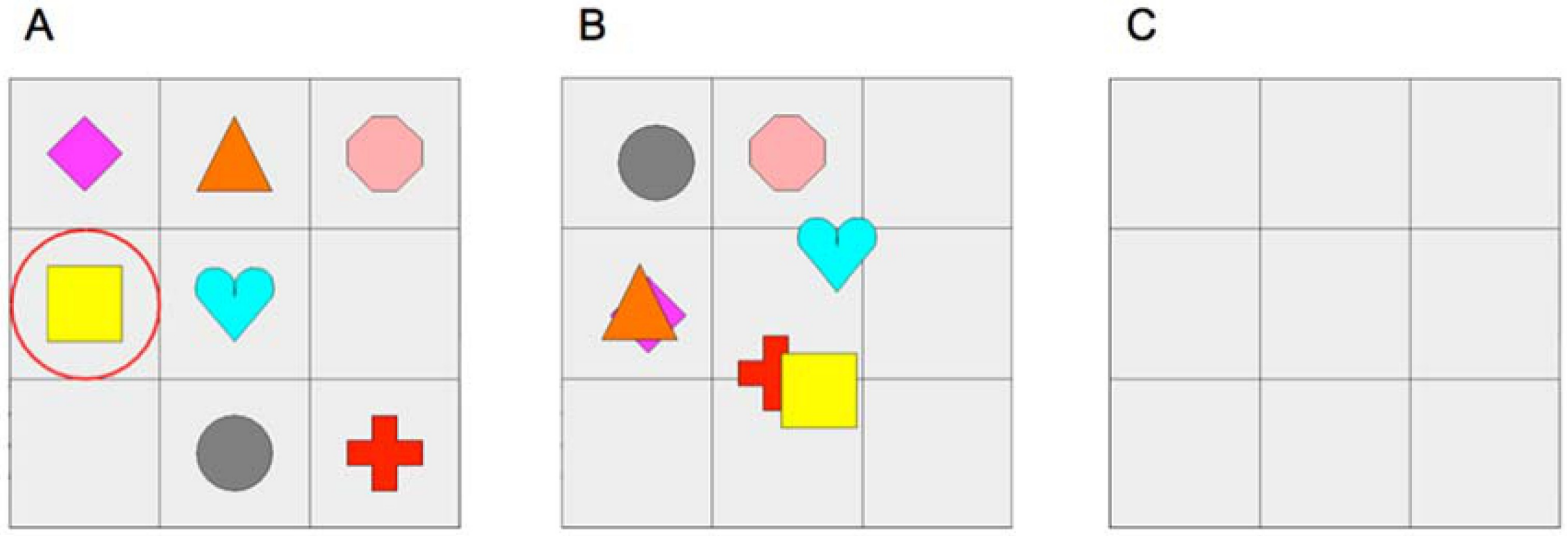
Track-It task. Screen captures depict (A) the beginning of a Track-It trial (target is yellow square); (B) the moment before the target and other shapes disappear; and (C) the end of the trial when the child must point to where they believe the target was before it disappeared.

To begin to address these issues by adding to the available measures that tap proactive control, we tested whether Track-It, a brief and child-friendly measure of selective sustained attention in children [27] also measures proactive control. In this task, children must track a target shape as it moves unpredictably among moving distractors and identify where it disappears (Fig 3). Successful performance likely requires anticipating that the target will disappear and engaging proactive control to track its location before it disappears. Engaging control only after realizing that the target is gone is unlikely to support recall of where it was when it disappeared given the presence of moving distractors and that the target could have disappeared in one of nine cells on the grid. For the same reasons, waiting until the target is incidentally seen moving somewhere on the screen would be unlikely to lead to success. Track-It can be completed in five minutes and involves brightly-colored, familiar shapes that children enjoy watching.

Across two experiments with two separate samples of children between the ages of 5 and 6 years (Ns = 33 and 62), we tested whether performance on Track-It predicts performance on existing developmental measures of proactive control. In Experiment 1, we investigated whether performance on Track-It predicts the tendency to proactively engage EF in a cued task-switching paradigm [20]. In Experiment 2, we examined whether Track-It predicts proactive control as measured by AX-CPT. Confirmatory findings would suggest that Track-It can be used to measure proactive control in children, and would raise interesting possibilities regarding the relationship between proactive control and selective sustained attention.

## Experiment 1

In Experiment 1, we examined whether performance on Track-It predicted proactive control in three conditions of a cued task-switching paradigm [20] that vary in the degree to which they sensitively measure children’s natural tendency to exercise proactive control. We used a version of Track-It with heterogeneous distractors (as in the heterogeneous condition in [27]), because performance on this version is thought to reflect predominantly endogenous processes while performance on a version with homogeneous distractors reflects both endogenous and exogenous processes. In the cued task-switching paradigm, the Proactive-Possible condition should sensitively measure individual differences in proactive control because children who are disposed to prepare will have time to do so and will have relatively fast RTs compared to those who do not prepare. Conversely, performance in the Proactive-Impossible condition should be relatively insensitive to individual differences in proactive control because children cannot proactively process the cue. Finally, in the Proactive-Encouraged condition there is an incentive to prepare, which may cause naturally reactive children to behave more proactively, and thus this condition should be less sensitive than the Proactive-Possible condition as a measure of individual differences in proactive control. Thus, if Track-It measures proactive control, it should more strongly predict RTs in the Proactive-Possible condition versus the Proactive-Impossible condition, and this pattern should hold when controlling for age and variability related to other task demands.

## Method

### Participants

Thirty-three 5-year-old children (M = 5.71 years, SD = .02, range = 5.19–5.99, female = 22) were recruited from a database of families who had previously indicated interest in participating in child development research. These children comprised a subset of 36 children recruited to participate in a study that also tested distinct questions related to metacognitive processes in the development of proactive control, the results of which were reported in [20]. Three children in the larger study sample did not complete Track-It and thus were excluded from the current study. Three additional children were recruited to participate in the larger study but were excluded due to uncooperativeness.

This research was approved by the Institutional Review Board at the University of Colorado. Parents provided written, informed consent to their children’s participation, and children provided verbal assent, all in accordance with IRB policy and approval.

### Procedure

Children first completed the three conditions of the cued task-switching paradigm followed by Track-It. Task order was fixed, minimizing variation between subjects in task performance due to differences in order [28].

#### Task-switching paradigm

The task-switching paradigm [20] (E-Prime 1.2; Psychology Software Tools, Pittsburgh, PA) was introduced as ‘The Santa Claus Game’, in which children were asked to help Santa prepare for next Christmas by sorting toys (i.e., the targets) by their shape or color. Three sets of shapes and colors were used (one for each condition): bear-car-blue-red, airplane-doll-green-orange, and train-horse-purple-pink. On each trial, children saw an 8cm x 8 cm target on the screen (e.g. a blue bear) along with a cue indicating the relevant sorting dimension. Children had to translate the cue and select the appropriate response using a response pad. Response options were indicated by 4 unidimensional pictures (e.g., a bear, a red patch, a car, and a blue patch) displayed on the screen (below the target) and also on the response pad right above the horizontally aligned response buttons.

Task cues were displayed within the black circle surrounding the target. The shape task was indicated by gray geometrical shapes and the color task was indicated by patches of different colors. On each trial, children first saw a fixation cross inside the black circle for 1000 to1200 ms (jittered inter-trial interval) followed by a gift box. After 1500 ms, the target replaced the box and remained on screen until a response was entered or for up to 10 seconds. The cue was also presented but, critically and as discussed in the Introduction, the timing of presentation varied across conditions (Fig 1). In each condition, children were instructed to look at the cue to determine how to sort the toy and to respond as quickly as they could.

Each condition began with the experimenter explaining the rules of each sorting game in turn. Explanations were accompanied by two demonstration trials in which the experimenter facilitated children’s sorting by asking them what game was being played and what button should be pressed. This was followed by four unfacilitated practice trials. Next the experimenter explained that children would play the two sorting games at the same time and that they needed to attend to the cue in order to know which game to play. This was followed by six demonstration trials and then eight practice trials. Corrective feedback was provided. In the test phase, children completed 62 trials compromised of start (2), switch (30) and no-switch (30) trials. Switch and no-switch trials alternated in a pseudorandom order. No corrective feedback was provided during the test trials.

#### Track-It task

In the Track-It task [27], children were presented with a 3 x 3 grid on a computer screen that was populated by seven shapes of different colors. The experimenter first guided children through a demonstration trial during which they were instructed that they needed to keep watching one of the shapes (target) as it moved across the grid in on random trajectory among the other moving colored shapes, and that all of the shapes would disappear and their job was to touch the screen where they last saw the target. Children then completed a memory check in which they were presented with four shapes in a 2 x 2 display, one of which was the target and the other three of which were among the distractors in the preceding trial. The position of the target in the display varied across memory check trials. Children were asked to identify which one of the shapes they were asked to watch. The memory check served as an inclusion criterion for scoring so that only trials on which children remembered which shape was the target were included in analyses. Children then completed 11 test trials, each of which was followed by a memory check. On each test trial, the target and distractors were randomly selected from a pool of nine unique colored objects. The speed of target and distracter objects was 600 pixels per frame at 30 frames per second, consistent with prior research involving the same age group [27]. Targets had to visit each of the nine cells and had to be positioned in the center of a given cell before disappearing. Trial duration was set to 10 seconds; however, actual trial length varied slightly to adhere to the motion restrictions [27].

## Results

### Data preparation and analytic approach

Proactive control in the cued task-switching paradigm was indexed via reaction times [20], and Track-It performance was indexed using the standard measure from this task of percent of trials on which children accurately identified the location of the target’s disappearance [27].

In our analyses we adopted a minimal RT trimming criterion of removing RTs faster than 200 ms to account for accidental button presses by children. This resulted in the removal of 3.7% of RTs. Beyond this minimal criterion we did not trim outliers at the shorter or longer end of the range. This approach was adopted because it is difficult if not impossible to make principled decisions about RT cutoffs, given the availability of multiple precedents in the literature that are equally justifiable. This increases researcher flexibility (also referred to as “researcher degrees of freedom”), which in turn can increase the rate of type 1 errors [29]. Additionally, when appropriate we used averages of individual’s median RTs to index central tendency, as they are more robust to extreme scores and appropriate given greater variability in child RT data [30].

We used R (R Core Team, 2012) to perform all statistical analyses and the lme4 package [31] to perform linear mixed effects analyses of the relationship between Track-It accuracy and proactive control as measured by the cued task-switching paradigm. We used linear mixed models because this analysis allowed us to account for variability due to trial type in our models (switch versus no-switch trials) while simultaneously accounting for dependency in our data given that trials were nested within subjects. Accounting for trial type variance in reaction times was expected to reduce error in our models and increase our ability to detect any effect of Track-It performance.

Because standalone measures of model fit are not produced in mixed effects models, we used nested model comparisons to isolate and test effects of predictors of interest. All estimates and standard errors corresponding to parameters of interest are reported in the text, along with likelihood ratio tests as recommended by [32] and demonstrated in [33]. Parameter estimates and standard errors for fixed effects from each set of models are provided in S1 Supporting Information. In traditional multiple regression analyses, all parameters in a model are estimated and tested via t-tests and p values, and are typically presented in a single table in the main text. However, in linear mixed model analyses, nested model comparison and likelihood ratio tests are instead used to isolate and test the predictor of interest, that is, by comparing a full model with the predictor to a reduced model without it. Thus the other predictors included in the compared models are not tested.

As fixed effects we included the following covariates in all models: age, trial type (switch vs. no-switch), and RT trial accuracy. Age was included because prior work has found that proactive control increases dramatically in childhood [18, 24, 21, 17]. Trial type was included as it is a known predictor of RT in cued task-switching paradigms generally and in this paradigm in particular [20]. Trial accuracy was included to account for any influence of accuracy on speed. As random effects we included intercepts for subjects to account for individual differences in subjects’ baseline RTs, and by-subject random slopes to account for individual differences in the subjects’ responses to the experimental manipulation [32]. For all models, we used Cook’s Distance to test for influential cases and all cases with Cook’s D values greater than three standard deviations above the mean Cook’s D for all observations were excluded. No model outliers were detected. Normal distribution of residuals was checked via inspection of a qq plot, histogram of the residuals, and a plot of standardized residuals against the predicted values, none of which revealed deviations from homoscedasticity or normality.

### Analyses

Descriptive analyses confirmed that on Track-It children showed near perfect memory for the target they were supposed to track in a given trial, and that ability to track the target was much poorer, as expected (Table 1). Taking memory into account, children’s tracking ability improved only trivially. A total of 11 test trials on which children misidentified the target they were supposed to track were discarded from our analyses. Skewness was -.83, with many children tending to show high accuracy given the specific parameter settings for this experiment, and kurtosis was .25. Descriptive analyses of the cued task-switching paradigm indicate that children were relatively accurate on test trials and that accuracy did not appear to vary significantly by trial type (Table 2). RTs for accurate trials were generally slower than for all trials.

**Table 1:** Descriptive statistics for Track-It memory and accuracy trial performance in Experiments 1 and 2.

	Experiment 1		Experiment 2	
	Mean	SD	Mean	SD
Memory trials	0.97	0.07	0.92	0.14
Accuracy	0.63	0.24	0.65	0.35
Accuracy (correct memory trials)	0.64	0.23	0.65	0.32

**Table 2:** Descriptive statistics for cued-task switching accuracy and RTs in Experiment 1.

Task	Index	Accuracy	SD	RT—all trials	SD	RT—accurate trials	SD
Possible							
	All trials	0.77	0.42	2906.56	1760.68	3023.99	1740.08
	No-switch trials	0.78	0.41	2809.16	1740.27	2919.92	1733.82
	Switch trials	0.75	0.43	3006.84	1776.71	3135.48	1740.93
Impossible							
	All trials	0.75	0.43	3171.69	1719.67	3359.77	1659.91
	No-switch trials	0.76	0.42	3108.62	1684.01	2991.92	1733.82
	Switch trials	0.74	0.44	3237.36	1754.34	3135.48	1740.93
Encouraged							
	All trials	0.68	0.47	2592.67	1563.52	2455.29	1397.11
	No-switch trials	0.69	0.46	2604.88	1607.16	2443.09	1447.42
	Switch trials	0.67	0.47	2580.14	1518.1	2468.29	1342.26

As predicted, Track-It accuracy was associated with faster RTs in the Proactive-Possible condition, controlling for median RT in the Proactive-Impossible condition. Partialing out variance related to performance in the Proactive-Impossible condition provides a more specific index of proactive control in the cued task-switching paradigm by controlling for other task demands. A full model including Track-It accuracy, median RT in the Proactive-Impossible condition and covariates was a better fit to the data than a reduced model that excluded Track-It accuracy, b = -1618.36 ± 629.79, χ^2^(1) = 6.02, p = .014. (Parameter estimates and standard errors for all variables included in all models are provided in Tables A—E in S1 Supporting Information.) The same analysis predicting RTs in the Proactive-Encouraged condition was not significant, b = -390.65 ± 431.41, χ^2^(1) = .80, p = .37. We found the same pattern using a multiple linear regression model predicting mean RT in the Proactive-Possible condition from Track-It accuracy on correct memory trials, controlling for mean RT in the Proactive-Impossible condition and age, b = -1189.16 ± 432 p = .008 (see Table F in S1 Supporting Information).

Track-It accuracy was a stronger predictor of RT in the Proactive-Possible condition than in the Proactive-Impossible condition. A full model including Track-It accuracy, condition, and their interaction, along with covariates, was a marginally better fit to the data than a reduced model that excluded the interaction term, b = -1287.19, ± 666.53, χ^2^(1) = 3.53, p = .06, indicating that the slope of the relation between Track-It accuracy and RT in the cued task-switching paradigm varied by condition. We found the same pattern using a simple linear regression model predicting the difference between condition means (RTs) from Track-It accuracy on correct memory trials (equivalent to a repeated-measures ANOVA), b = -813.7, 393.9, p = .043 (see Table G in S1 Supporting Information).

We followed this analysis with an investigation of the simple relations between Track-It and RT for each condition, and found that Track-It’s predictive strength varied across the three cued task-switching conditions in ways that were generally consistent with our task analysis. For each condition, we compared full models that included Track-It accuracy and covariates to reduced models that excluded Track-It accuracy. Track-It accuracy predicted faster RTs in the Proactive-Possible condition, b = -1885.11 ms ± 649.76, χ^2^(1) = 7.50, p = .006, but not in the Proactive-Impossible condition, b = -478.08 ms ± 619.65, χ^2^(1) = .59, p = .44. Track-It also did not predict trial RT in the Proactive-Encouraged condition, b = -686.81 ms ± 496.68, χ^2^(1) = 1.80, p = .18. An exploratory analysis indicated that Track-It accuracy tended to be a stronger predictor of RT in the Proactive-Possible condition than in the Proactive-Encouraged condition, such that the interaction between condition and accuracy was marginally significant, b = 1039.20, ± 582.38, χ^2^(1) = 3.07, p = .08. The same analysis comparing the Proactive-Impossible and Proactive-Encouraged conditions was not significant, b = 243.08, ± 489.15, χ^2^(1) = .25, p = .62, indicating that Track-It did not differentially predict RTs across these two conditions.

## Discussion

Consistent with our hypothesis that Track-It measures proactive control, Track-It accuracy was more strongly associated with fast RTs in the Proactive-Possible condition versus the Proactive-Impossible condition, controlling for other aspects of the task (switching demands, accuracy), age, and individual differences in baseline RT. Track-It accuracy significantly predicted fast RTs in the Proactive-Possible condition but not Proactive-Impossible condition.

This experiment provides the first evidence that Track-It, a relatively quick and easy-to-administer task developed for use with young children, may measure proactive control. However, it is possible that, in spite of our efforts to control for other aspects of performance, the observed relationship reflects some other cognitive process tapped by both tasks. Although Track-It accuracy was more strongly related to RT in the condition that was designed to allow children to use proactive control than the condition that was designed to prevent the use of proactive control, it could be that the slopes differed as a function of some other uncontrolled factor that varied between the two conditions. Finding that Track-It correlates with other established measures of proactive control would thus provide more compelling, converging evidence. We tested this possibility in Experiment 2.

## Experiment 2

Experiment 2 examined the relation between Track-It and another established, specific measure of proactive control, AX-CPT. If Track-It predicts performance on AX-CPT, this would further suggest that it can be used as a measure of proactive control.

## Method

### Participants

Sixty-four 5- and 6-year-old children (M = 5.45 years, SD = .5, range = 4.92–6.08, female = 34) completed both Track-It and the AX-CPT. An additional 9 children were excluded from analyses because of uncooperativeness and/or failure to follow directions (Track-It n = 3; AX-CPT n = 5) or experimenter error (n = 1). Children were recruited as part of a larger study that also tested questions related to the costs and benefits of transitioning from primarily reactive to increasingly proactive control; only results relating to the question of whether performance on Track-It and AX-CPT are related are reported in the present manuscript.

### Procedure

As in Experiment 1, the order of the tasks was fixed; children completed the AX-CPT, Track-It, and two tasks that were unrelated to the goals of the current study (indexing cognitive flexibility and cognitive monitoring).

#### AX continuous performance task (AX-CPT)

Children completed a touchscreen-based, child-friendly version of the AX-CPT [18], where cues were presented as cartoon characters, and targets were presented as cartoon objects. Children were asked to remember character preferences for different objects, and make choices based on them. For example, children were told, “SpongeBob likes watermelon, so press the happy face when you see SpongeBob and then the watermelon,” and, “Blue doesn't like the slinky, so press the sad face when you see Blue and then the slinky.” Children were instructed to respond with a target response (e.g., a happy face) whenever the “A” context cue (SpongeBob) was followed by an “X” probe (watermelon), and to respond with a non-target response to all other cue-probe sequences (AY, BX, BY). Stimuli were counterbalanced across children.

Prior to test trials, children answered questions about the task rules and completed practice trials. During the rule verification phase, the experimenter asked participants to indicate the correct response for each cue–probe pair. If a participant did not state the correct rule for a cue-probe pair, the experimenter repeated the relevant rule (“Remember, when you see [A, B] and then you see [X, Y], tap this button [taps appropriate button] as quickly as you can!”), and asked the child if s/he wanted to try again. After the rule verification phrase, children completed 7 practice trials. During each trial, a cue was presented for 500 ms, followed by a 1.25 s delay period, and a subsequent probe. The response window following probes was adaptive, such that the allowable response time was initially set to 6 s, and was subsequently adjusted to 150% of the child’s mean RT on the previous eight trials. If a child did not respond within the allotted time, the image of a sleeping alarm clock appeared, and the experimenter stated, ‘‘Oops! Too slow. Try to tap the button faster next time.”

Children completed 120 test trials presented in four 30-trial blocks, where 70% of trials were target (AX) trials, and 30% were non-target trials (AY, BX, BY, appearing in equal proportion). Stimulus, probe, and response window durations were set as in practice trials. Two out of the four test blocks included an additional manipulation: during the delay interval between presentation of the cue and the probe, full-screen images of natural landscapes (e.g., mountains, a beach, a waterfall) were displayed. These images were intended to be distracting, making it more difficult for children to maintain cue information across the delay period. During the 1.25 s delay period between cue and probe presentation, children saw either one or two consecutively-presented landscape images presented for 500 ms. No image was presented more than once during the experiment. In the other two blocks, children viewed a fixation cross during the cue-probe delay interval.

#### Track-It task

Track-It [27] administration and inclusion criteria were identical to those in Experiment 1.

## Results

### Data preparation and analytic approach

Track-It performance was indexed as in Experiment 1. We generated two measures of proactive control on AX-CPT, based on accuracy and reaction time, that have been used in prior work [18, 21]. These measures index children’s relative performance in BX trials, where proactive control can support performance, and AY trials, where proactive control can interfere with performance. Our accuracy measure was the proportional difference in AY versus BX errors using the formula (AY errors–BX errors)/(AY errors + BX errors). For trial-types where no errors were made, a correction was applied (as in past work using this measure; Braver et al., 2009), calculated as (error—0.5)/(frequency of trial-type trials + 1). Our RT measure indexed child slowing in correct AY relative to correct BX trials, calculated as within-subject z-scored mean AY trial RT less within-subject z-scored mean BX trial RT [21]. Two children did not respond correctly to any BX trials, and were therefore excluded from this RT-based analysis.

AX-CPT responses made < 200 ms after the presentation of the probe were removed from the analysis, resulting in the exclusion of < 1% of all trials. AX-CPT block order (distractor block first versus second) did not predict differences in proactive control indices (ps > .5). To test whether the inclusion of a distracting image influenced proactive control, reaction-time and accuracy-based measures of proactive control were compared across distractor and no-distractor blocks. No differences were found for either measure (ps > .3). This result, along with the finding of similar performance patterns in this version as in versions without the distractor block (e.g., slower RTs/lower accuracy on AY and BX trials; [18, 21]) suggests the validity of the task as measure of proactive control was not compromised by the presence of distractor blocks. Therefore, proactive control measures were based on RTs averaged across all blocks. To account for individual differences in processing speed, within-participant z-scores were calculated for each trial type (AX, AY, BX, BY). For all correct trials, the difference between trial RT and overall participant mean RT was divided by the participant’s SD. The resulting standardized scores were averaged within participants, within trial type.

For all models, outlying observations generating Cook’s D values greater than three standard deviations above the mean Cook’s D for all observations were excluded. This resulted in the exclusion of no more than two observations from any model.

### Analyses

As in Experiment 1, children were almost always able to identify the target they were supposed to track in a given trial (> 90% accuracy, Table 3). Children’s tracking accuracy was not driven by their memory for the target, as rates were comparable before and after trials in which the target was not identified at post-test were excluded (n = 61). Skewness was -.70 and kurtosis was .84. We also found evidence that Track-It is a reliable measure. We assessed split-half reliability by combining across our two samples, and testing the correlation between odd and even trials (the order of which was random within participants). The Pearson correlation was r(94) = .73, p < .001. Correcting this estimate using the Spearman-Brown prophecy formula yielded an adjusted r of .84, indicating very good reliability. Descriptive analyses of AX-CPT performance are given in Table 3. As predicted, children were more accurate and faster to respond in trials where both the cue and probe were valid or invalid (AX and BY trials), relative to trials where either the cue or probe was invalid (AY and BX trials).

**Table 3:** Descriptive statistics for AX-CPT performance in Experiment 2.

	Trial Type			
	AX	AY	BX	BY
Mean Accuracy	86.6 (.09)	64.4 (.19)	61.7 (.26)	81.2 (.18)
Mean RT (ms)	1366.0 (368.64)	1621.0 (402.44)	1563.0 (615.52)	1414.0 (421.65)

Note: Parentheses indicate standard deviation.

To test whether children’s Track-It accuracy related to proactive behavior on the AX-CPT, we generated linear models where AX-CPT indices of proactive control were predicted by Track-It accuracy and child age. No interactions were entered in the models. Children’s performance on the AX-CPT accuracy-based measure of proactive control was positively related to their accuracy on Track-It, b = 0.55; F(1, 59) = 7.59; p = .008, η^2^_p_ = 0.11. The AX-CPT RT-based measure of proactive control showed a trend relationship with Track-It accuracy in the expected direction, though this relationship did not meet statistical significance criteria, b = .52; F(1, 57) = 2.62; p = .11, η^2^_p_ = 0.04.

## Discussion

Consistent with our expectation that Track-It measures proactive control, and with the findings of Experiment 1, Track-It predicted proactive control as measured by reduced accuracy on AY relative to BX trials on AX-CPT. This provides further evidence that the relation between Track-It and these proactive control tasks is not likely due to their tapping some other common, unaccounted for cognitive skill. As in Experiment 1, these findings held when controlling for age.

The finding that the Track-It was significantly correlated with accuracy but not RT on AX-CPT is consistent with the possibility that accuracy is a more sensitive measure of proactive control in this age group than RT, which can be highly variable in children [30]. Nevertheless, the correlational trend between Track-It and the AX-CPT RT measure was in the expected direction, providing some converging evidence across two indices of proactive control in AX-CPT that Track-It measures proactive control.

## General discussion

We present the first evidence that Track-It, a quick, easy-to-administer task developed to assess selective sustained attention in children can also serve as a measure of proactive control. Across two experiments involving two separate established measures of proactive control, accuracy on Track-It significantly predicted performance, controlling for age and other task factors.

These relationships are unlikely to reflect other factors such as alertness or motivation, which could lead some children to perform better across these tasks as well as any others. Such factors would be expected to improve performance across all trials and conditions (e.g., including the Proactive Impossible condition in the Task-Switching paradigm, and AY trials in AX-CPT), whereas the patterns we found were specific to profiles of proactive control, and these patterns held when controlling for other possible individual differences. The current study thus represents a first step in validating Track-It as a measure of proactive control by finding performance converges with that of two established proactive control measures, and shows specificity to proactive profiles; however, more research is needed to further assess convergent and divergent validity. Future research could further test whether these measures tap a common underlying factor that is distinct from other factors, for example, by conducting latent variable analyses to assess whether performance on proactive control and selective sustained attention tasks are explained by a single underlying factor that is distinct from other aspects of executive functioning [25].

The methods of the current study were correlational, leaving open interesting possibilities regarding how proactive control and selective sustained attention may be related. Engaging control in anticipation of needing it could support the ability to selectively attend to stimuli in the face of distraction. For example, anticipating the need to make a response and proactively maintaining information about the target in Track-It could support selective sustained attention to the target, as we hypothesized. Conversely, selectively attending to stimuli in the face of distraction could support the ability to anticipate the need to make a response and to proactively maintain relevant information. In this case, the correlations in the current studies could also reflect the role of selective sustained attention in performance on both Track-It and established measures of proactive control. Future work could test such possibilities by manipulating proactive control experimentally and testing for effects on selective sustained attention, and vice versa. For example, adoption of a proactive strategy could be encouraged (e.g., as in [20]) and children’s encoding or attention to task-relevant and irrelevant stimuli features could be assessed, with the expectation that if proactive control influences selective sustained attention then children who engage more proactive control will be more likely to selectively attend to and encode task-relevant aspects of the stimuli. Conversely, selective sustained attention could be manipulated and selective effects on measures of proactive control could be assessed.

More generally, such possibilities highlight that proactive control and selective sustained attention may be mutually supporting. They may also rely on common mechanisms, such as goal maintenance, with sustained activation of goal information supporting selective attention [33, 34] and anticipatory control across time. Future work could test this possibility by manipulating the need for goal maintenance (e.g., by inducing goal neglect by providing goal cues on a portion of trials, thereby making goal maintenance redundant; [35] and testing effects on proactive control and selective sustained attention.

The current study opens new avenues for examining the nature and development of proactive control in childhood and its relationship to other key skills. While it is well established that EF develops rapidly in early childhood, the discovery that there is age-related variability in the temporal dynamics of its engagement is recent, and there is much to explore regarding the implications of this developmental transition. For example, how might the addition of proactive control to children’s cognitive repertoires enable more adaptive functioning? What are the costs and benefits of developing proactive control [16, 36]? How do children develop sensitivity to such costs and benefits to adaptively coordinate distinct forms of EF (e.g., [37])? And how much of the relationship between EF and various positive outcomes to which it has been linked in childhood is explained by proactive control? Adding Track-It to the pool of available childhood measures of proactive control will allow researchers to address these and other key questions.

## Conclusion

The current study reports converging evidence that Track-It, an easy-to-administer task developed to measure selective sustained attention in children, can also serve as an index of proactive control. Using Track-It alone or in conjunction with other childhood measures of proactive control can advance knowledge on how it relates to EF and associated outcomes.

## Supporting information

S1 Supporting Information Tables A—F.
(DOCX)
