# Supplementary material for: Getting ready to use control: Advances in the measurement of young children’s use of proactive control
Source: PLoS One. 2017 Apr 18;12(4):e0175072. doi: 10.1371/journal.pone.0175072 (PMC5395143; doi:10.1371/journal.pone.0175072)
Supplement: S1 Supporting Information — Tables A—F. (DOCX) [file pone.0175072.s001.docx]

Note: All models include random intercepts for subjects.

Table A

Fixed effect parameter estimates from linear mixed models testing whether Track-It accuracy predicts reaction time in the proactive-possible condition of the switching paradigm. “------“ indicates parameter was not estimated in the model.

|  | Reduced Model | | Full model | |  |  |
| --- | --- | --- | --- | --- | --- | --- |
| Predictor | Estimate | SE | Estimate | SE | χ^2^ | *p* |
| Intercept | 3704 | 3540 | 1587 | 3334 |  |  |
| Impossible med RT | .45 | .19 | .35 | .18 |  |  |
| Age | -32 | 50 | 17 | 50 |  |  |
| Trial type | 199 | 66 | 199 | 66 |  |  |
| Trial accuracy | 469 | 88 | 469 | 88 |  |  |
| Track-It | ------ | ------ | -1618 | 629 | 6.02 | .014 |

Table B

Fixed effect parameter estimates from linear mixed models testing whether the interaction between Track-It accuracy and Condition (Possible vs. Impossible) predicts reaction time on the cued task-switching paradigm. “------“ indicates parameter was not estimated in the model. Includes random slopes modeling effect of condition for each subject.

|  | Reduced Model | | Full model | |  |  |
| --- | --- | --- | --- | --- | --- | --- |
| Predictor | Estimate | SE | Estimate | SE | χ^2^ | *p* |
| Intercept | 2435 | 2660 | 2406 | 2659 |  |  |
| Age | 6.25 | 38 | 6.67 | 38 |  |  |
| Trial accuracy | 176 | 47 | 175 | 47 |  |  |
| Trial type | 101 | 38 | 101 | 38 |  |  |
| Track-It accuracy | -956 | 473 | -937 | 474 |  |  |
| Condition | -261 | 159 | -261 | 151 |  |  |
| Track-It X Condition | ------ | ------ | -1287 | 666 | 3.53 | .06 |

Table C

Fixed effect parameter estimates from linear mixed models testing whether Track-It accuracy predicts reaction time in the proactive-possible condition of the switching paradigm. “------“ indicates parameter was not estimated in the model.

|  | Reduced Model | | Full model | |  |  |
| --- | --- | --- | --- | --- | --- | --- |
| Predictor | Estimate | SE | Estimate | SE | χ^2^ | *p* |
| Intercept | 5453 | 3741 | 1334 | 3628 |  |  |
| Age | -38 | 54 | 21 | 52 |  |  |
| Trial type | 199 | 66 | 199 | 66 |  |  |
| Trial accuracy | 470 | 88 | 469 | 88 |  |  |
| Track-It | ------ | ------ | -1885 | 649 | 7.49 | .006 |

Table D

Fixed effect parameter estimates from linear mixed models testing whether Track-It accuracy predicts reaction time in the proactive-impossible condition of the switching paradigm. “------“ indicates parameter was not estimated in the model.

|  | Reduced Model | | Full model | |  |  |
| --- | --- | --- | --- | --- | --- | --- |
| Predictor | Estimate | SE | Estimate | SE | χ^2^ | *p* |
| Intercept | 5121 | 3216 | 4071 | 3464 |  |  |
| Age | -29 | 46 | -14 | 50 |  |  |
| Trial type | 145 | 66 | 145 | 66 |  |  |
| Trial accuracy | 440 | 87 | 443 | 87 |  |  |
| Track-It | ------ | ------ | -478 | 619 | .58 | .44 |

Table E

Fixed effect parameter estimates from linear mixed models testing whether Track-It accuracy predicts reaction time in the proactive-encouraged condition of the switching paradigm. “------“ indicates parameter was not estimated in the model.

|  | Reduced Model | | Full model | |  |  |
| --- | --- | --- | --- | --- | --- | --- |
| Predictor | Estimate | SE | Estimate | SE | χ^2^ | *p* |
| Intercept | 2494 | 2674 | 995 | 2789 |  |  |
| Age | 3.01 | 39 | 24 | 40 |  |  |
| Trial type | -39 | 61 | -39 | 61 |  |  |
| Trial accuracy | -511 | 70 | -508 | 70 |  |  |
| Track-It | ------ | ------ | -686 | 496 | 1.80 | .18 |

Table F

Linear regression testing whether Track-It accuracy predicts difference in reaction time between proactive-possible and impossible condition (comparable analysis to Tables B and C but not accounting for variance due to trial type and age, and including accurate RTs only).

| Predictor | Estimate | SE | t | *p* |
| --- | --- | --- | --- | --- |
| Intercept | 309 | 313 | .98 | .32 |
| Track-It | -813 | 393 | -2.06 | .04 |

Table G

Multiple linear regression testing whether Track-It accuracy predicts reaction time in proactive-possible condition (comparable analysis to Table A but not accounting for variance due to trial type, and including accurate RTs only).

| Predictor | Estimate | SE | t | *p* |
| --- | --- | --- | --- | --- |
| Intercept | 3392 | 722 | 4.69 | <.001 |
| Mean RT Proactive-Impossible Condition | .39 | .12 | 3.17 | .002 |
| Age | -14 | 5.27 | -2.74 | .008 |
| Track-It | -1189 | 432 | -2.75 | .008 |
